# Automatic categorization of self-acknowledged limitations in randomized controlled trial publications

**DOI:** 10.1016/j.jbi.2024.104628

**Published:** 2024-03-26

**Authors:** Mengfei Lan, Mandy Cheng, Linh Hoang, Gerben ter Riet, Halil Kilicoglu

**Affiliations:** a School of Information Sciences, University of Illinois Urbana-Champaign, 501 Daniel Street, Champaign, 61820, IL, USA; b Department of Biological Sciences, Binghamton University, 4400 Vestal Parkway East, New York City, 13902, NY, USA; c Faculty of Health, Amsterdam University of Applied Sciences, Tafelbergweg 51, Amsterdam, 1105 BD, The Netherlands

**Keywords:** Self-acknowledged limitations, Randomized controlled trials, Reporting quality, Natural language processing, Text classification, Large language models

## Abstract

**Objective:**

Acknowledging study limitations in a scientific publication is a crucial element in scientific transparency and progress. However, limitation reporting is often inadequate. Natural language processing (NLP) methods could support automated reporting checks, improving research transparency. In this study, our objective was to develop a dataset and NLP methods to detect and categorize self-acknowledged limitations (e.g., sample size, blinding) reported in randomized controlled trial (RCT) publications.

**Methods:**

We created a data model of limitation types in RCT studies and annotated a corpus of 200 fulltext RCT publications using this data model. We fine-tuned BERT-based sentence classification models to recognize the limitation sentences and their types. To address the small size of the annotated corpus, we experimented with data augmentation approaches, including Easy Data Augmentation (EDA) and Prompt-Based Data Augmentation (PromDA). We applied the best-performing model to a set of about 12K RCT publications to characterize self-acknowledged limitations at larger scale.

**Results:**

Our data model consists of 15 categories and 24 sub-categories (e.g., Population and its sub-category DiagnosticCriteria). We annotated 1090 instances of limitation types in 952 sentences (4.8 limitation sentences and 5.5 limitation types per article). A fine-tuned PubMedBERT model for limitation sentence classification improved upon our earlier model by about 1.5 absolute percentage points in F_1_ score (0.821 vs. 0.8) with statistical significance (*p* < .001). Our best-performing limitation type classification model, PubMedBERT fine-tuning with PromDA (Output View), achieved an F_1_ score of 0.7, improving upon the vanilla PubMedBERT model by 2.7 percentage points, with statistical significance (*p* < .001)

**Conclusion:**

The model could support automated screening tools which can be used by journals to draw the authors’ attention to reporting issues. Automatic extraction of limitations from RCT publications could benefit peer review and evidence synthesis, and support advanced methods to search and aggregate the evidence from the clinical trial literature.

## 1. Introduction

Biomedical publishing has been transformed in recent years, spurred in part by the COVID-19 pandemic [1]. There has been a sharp rise in non-peer-reviewed preprint publications [2], and the speed with which these studies have been conducted and published has raised concerns in scientific and lay press about the methodological and reporting quality of COVID-19 research [3–6]; some publications in prominent journals were eventually discredited or retracted [1]. Such issues, of course, are not new or limited to COVID-19 research, or even to biomedical science. Problems in study design, execution, data analysis, and reporting affect the validity and applicability of the findings in any field. It is essential for researchers to acknowledge potential weaknesses and biases of their studies (i.e., its limitations) and discuss their magnitude when publishing their findings [7,8]. Recognizing and discussing limitations is essential for scientific progress, as they help the reader contextualize the study, understand its findings, and assess the credibility of these findings [7]. Limitation discussions could also reveal future research directions and the caveats that need to be considered when incorporating the new findings into scientific evidence [7,9,10].

Study protocols are increasingly accessible, making it possible for experts to decide on the important study limitations themselves if authors are open about all discrepancies between study plan and execution; however, publications should also be optimally informative for readers who are not seasoned experts in a particular research field and frank limitations sections will probably remain important [11].

Randomized controlled trials (RCTs) are a cornerstone of clinical medicine and provide the most robust methodology to generate evidence on the effectiveness of therapeutic interventions [12,13]. To fully realize their potential for informing patient care and health policies, they need to observe high methodological and reporting quality standards [12–14]. However, this is often not the case, leading to low-quality evidence and significant research waste [14]. One common reporting problem in RCT publications is that authors often do not properly acknowledge the limitations of their study [7,11,15,16], making it difficult for stakeholders (peer reviewers, journal editors, systematic reviewers, clinicians, policymakers) to contextualize the findings and assess their trustworthiness [7]. CONSORT reporting guidelines for RCT publications [12,17] recommends reporting of limitations in RCT publications and states that “trial limitations, addressing sources of potential bias, imprecision, and, if relevant, multiplicity of analyses” [12] should be discussed in Discussion sections. However, reporting of limitations has been found to be inadequate [11,18], similar to other checklist items [19–21]. Automated screening tools can reduce the time and effort for manual checks in journals, provide rapid reporting quality assessments of preprints, and raise awareness of poor reporting practices among researchers [22–24], leading to gradual improvements in RCT reporting. Natural language processing (NLP) techniques can underpin such screening tools [23–26].

In prior work, we developed NLP models for recognizing sentences discussing self-acknowledged limitations (SALs) in clinical publications [27]. We compared several models, including a rule-based approach, a logistic regression classifier, and a SVM classifier, and experimented with self-training to address the data scarcity problem. The rule-based method performed reasonably well (0.80 F_1_ score and 0.915 accuracy). We used this method to study the impact of peer review on discussion of study limitations [28], finding support for the idea that editorial processes lead to more self-acknowledgment of study limitations. This method was later incorporated into a pipeline of tools for screening COVID-19 preprints for transparency and reproducibility [23,24]. While automatically identifying limitation sentences are useful as a simple reporting check (i.e., have limitations been acknowledged?), extracting the types of limitations reported could help in: (a) more precisely contextualizing a study’s findings and assessing their credibility; and (b) better understanding the common types of problems in large sets of research studies, including RCTs, to answer meta-research questions. In this study, we extend our prior work [27] by extracting self-acknowledged limitation types from RCT publications. We make the following contributions:

1. We improve our SAL sentence recognition model by fine-tuning the PubMedBERT model [29]
2. We develop a fine-grained data model of limitation categories, taking related work [30] as our starting point.
3. We manually annotate a corpus of 200 RCT publications with SAL categories at fine granularity.
4. We fine-tune the PubMedBERT model for multi-label sentence level classification of SAL types.
5. We experiment with prompt-based data augmentation with the help of a large language model to address the data imbalance and scarcity problem.
6. We analyze the model output on a large corpus of RCT publications to describe the commonly reported limitations of RCTs.

Our results show that annotating SAL types and achieving good inter-annotator agreement (IAA) is challenging. Our NLP model shows that it is possible to recognize SAL types at coarse granularity, while experiments with finer granularity yield more modest results. Prompt-based data augmentation improves NLP model performance. Sample size and population-related limitations are the most commonly discussed.

**Table:** Statement of significance.

Problem	It is essential for researchers to acknowledge limitations of their studies, so that their findings can be contextualized. It is unknown whether researchers do this consistently and what types of limitations they report.
What is already known?	Some NLP research has explored automatic identification of self-acknowledged limitation sentences in biomedical publications.
What this Paper Adds?	We improve sentence classification models for self-acknowledged limitations. We develop a fine-grained data model of limitation types for RCT publications and annotate a corpus. We fine-tune a NLP model to recognize limitation types, incorporating prompt-based data augmentation. We assess limitation reporting in a set of about 12K RCT publications.

## 2. Related work

Existing literature discusses the importance of acknowledging limitations and their potential implications for the interpretation of findings [7,9,11,15,16,30,31]. Limitation discussion is also viewed positively, as it can inform future studies that address these limitations and produce higher quality studies [9–11]. Studies on SALs in biomedical literature found that authors often fail to discuss them. The acknowledgment of limitations was found to be the most problematic among 34 items of manuscript quality at submission [15]. Limitation reporting was also quantified, estimates ranging from 17% [7] to 70% [27] and 73% [11]. Reporting guidelines, such as CONSORT for RCTs [17] and ARRIVE for preclinical studies [32], recommend discussion of limitations.

Automated tools to recognize SALs can support manuscript screening, improving the reporting quality of published literature, and scale up meta-research investigations on limitations. Aside from our prior work mentioned above [27], there is little NLP research in recognizing SALs in the biomedical literature. One study investigated limitation reporting in COVID-19 publications using simple keyword searches and manual analysis [33]. Another study focused on recognizing sentences describing challenges and directions in COVID-19 publications as the basis of a discovery search engine, where the challenge category overlaps with limitations [10]. To the best of our knowledge, no NLP research for identifying the types of SALs reported in scientific publications has been previously reported.

On the other hand, there is a significant body of literature on natural language processing for RCT publications. Most of this literature has focused on extracting PICO classes (Population, Intervention, Comparator, Outcome) to assist systematic reviews and facilitate evidence-based medicine, often using sentence classification [34–40] and named entity recognition [40–44]. Some research focused on classifying RCT abstract sentences along IMRaD (Introduction-Methods-Results-Discussion) categories [37,39,45]. Other recent studies investigated extraction of information relevant to methodological assessment of RCT studies, such as risk of bias information [46], study characteristics (e.g., sample size) [47,48], and CONSORT checklist items [22,49,50]. Recent state-of-the-art models have relied on domain-specific Transformer-based, pretrained language models [51].

To enable NLP model development and validation, a large amount of high-quality labeled data is often needed. Labeling such data, especially in biomedical domain, is challenging, because annotation is time-consuming and requires significant domain expertise. Methods to address small sample sizes have been proposed, often studied under the umbrella of weak supervision and data augmentation. Weak supervision attempts to use domain knowledge and subject matter expertise to assign somewhat noisy labels to unlabeled data [52]. Data augmentation generates realistic examples from a limited number of existing examples [53]. For example, simple transformations of individual sentences (e.g., synonym replacement, random deletion/insertion) have been used to improve modeling accuracy with small datasets [54,55]. Given that such modifications may distort the original meaning of the text, recent research studies have used large language models to synthesize more meaningful sentences [56–59].

## 3. Material and methods

### 3.1. Improving SAL sentence classification

We fine-tuned the PubMedBERT model [29] from HuggingFace’s model repository (microsoft/BiomedNLP-PubMedBERTbase-uncased-abstract-fulltext) on the limitation sentence dataset reported in prior work [27]. This dataset consists of 2,257 sentences, 467 (20.7%) of which include SALs. As the input representation for the model, we concatenated the target sentence, the top section header (e.g., Discussion), and the innermost section header (e.g., Study Limitations) and fed the [CLS] token representation generated by PubMedBERT to a linear layer for binary classification. Binary cross-entropy loss is used as the loss function. Detailed experimental settings are provided in Appendix B.

### 3.2. Data collection for SAL type annotation

We collected a dataset of 200 RCT articles for annotation. A subset of the articles (52) came from our previous studies on limitation sentence classification [27] and CONSORT sentence classification [22]. Only those articles containing sentences manually labeled as limitation sentences were included. The rest of the RCT articles were sampled from PubMed Central Open Access Subset (PMC-OA) using a modified version of Cochrane’s sensitivity and precision-maximizing query for RCTs as the search strategy. We split the downloaded RCT articles into sentences using the NLTK sentence tokenizer and identified the section to which a sentence belongs using a section recognizer. Then, the SAL sentence classifier based on PubMedBERT fine-tuning (described above) was used to identify SAL sentences from the abstract and discussion- and limitation-related sections, indicated by the keywords *discussion*, *limitation*, *weakness*, *conclusion*, *caveat*, *shortcoming*, and *drawback* in the header, consistent with previous work [27]. One of the authors (HK) manually assessed the accuracy of the sentence classifier predictions to verify that only RCT articles with at least one SAL sentence were included in the corpus.

### 3.3. Data model for SAL types

To create the data model for annotation, we started with the limitation categorization presented in a recent investigation of manual therapy trials [30]. Their categorization consists of 12 top-level categories (e.g., Blinding, Sample Size, Inadequate Control, Compromised Generalization) and 38 sub-categories (e.g., Underpowered Study, Convenience Sampling, and Recruitment Less Than Expected for the Sample Size category). After a test annotation of 10 articles, we recognized the need to simplify and adapt the categorization, as some categories related to specific characteristics of manual therapy trials (e.g., Therapist Profile) and some categories seemed difficult to reliably differentiate (e.g., Intervention vs. Compromised Generalization due to Intervention). As a result, two authors (HK and GtR) redesigned the data model in several iterations. The final data model consists of 15 top-level categories and 24 sub-categories. The data model and the definitions of the categories are provided in Table 1 and annotation examples in Appendix Table A.1.

### 3.4. SAL type annotation

We used the BRAT tool [60] for annotating SAL types. We developed annotation guidelines and refined the instructions over the course of the annotation (provided as Supplementary Material). We pre-annotated limitation sentences. The annotators were instructed to label spans indicating specific SAL types in these sentences only. They were also instructed to use sub-categories, when possible. Top-level categories could be used when the sub-categories did not adequately describe the limitation type. Five annotators (authors of this paper) were involved in annotation: a research methodology/epidemiology expert (GtR) and a biomedical informatics/NLP expert (HK), both of whom also designed our previous study on SALs [27], two Ph.D. students trained in biomedical NLP (ML and LH), and an undergraduate student in biological sciences (MC) with experience in biomedical literature. 50 out of 200 articles were annotated by all annotators over two stages, and IAA was calculated for each stage. The annotations were reconciled by HK. The rest of the articles were split into five parts and assigned to individual annotators. The resulting annotations were checked for correctness and consistency by HK. We calculated IAA in two ways. First, we calculated sentence-level IAA using Krippendorff’s *α* [61]. We used this measure because it accommodates more than two annotators and missing annotations. Agreement at both the top level and the fine-grained level were considered. We used MASI [62] as the distance metric for Krippendorff’s *α*. Second, we used Cohen’s *κ* [63] to calculate pairwise agreement at the token level. These calculations help us better understand the annotation challenges and design NLP models.

### 3.5. SAL type categorization

We formulated the task of identifying SAL types discussed in an RCT publication as multi-label sentence classification, despite associating these types with spans in annotation. This is due to a couple of reasons. First, we observed that spans indicating SAL types were very heterogeneous, ranging from short noun phrases which look like typical named entities to entire sentences. Secondly, IAA for span annotation was found to be low (results below), probably due to this heterogeneity. Because the goal of SAL type categorization is ultimately to understand which limitation types are described in an article, not finding the exact spans indicating them, a sentence multi-label classification approach was deemed appropriate. We converted and consolidated span-level annotations to sentence-level annotations. As our classification scheme, we experimented with both the top-level and the fine-grained categorization. Note that in fine-grained categorization, top-level categories are still considered, because it is possible that some sentences only have top-level labels. The dataset was split into training/development/test sets of 120, 40, and 40 articles, respectively.

As the baseline model for SAL type categorization, we also fine-tuned the same PubMedBERT model [51] using the target sentence prepended with the section headers as input. Similarly, in this case, the representation for the [CLS] token for the target sentence is fed into a multi-layer perceptron (MLP) and a softmax layer that calculates the probability distribution of each label for the target sentence. We then apply dynamic thresholding, which uses different probability thresholds for each label. The optimal threshold for each label is determined based on the label-specific F_1_ score on the development set. Cross-entropy loss is used as the loss function. Experimental settings for the models are provided in Appendix B.

#### 3.5.1. Prompt-based sentence augmentation

Our annotation yielded an imbalanced dataset with few examples for some categories, including some top-level categories. To address these shortcomings, we used data augmentation to synthesize novel samples for the less frequent classes. As our primary data augmentation method, we adapted the PromDA method (Prompt-Based Data Augmentation) [59], which is built upon the *T5-Large* encoder–decoder model [64]. It keeps the entire pre-trained T5 model frozen, prepends additional soft prompts (i.e., a sequence of continuous and trainable vectors) in each layer of the model, and tunes the soft prompts only [65]. Soft prompts are pre-trained using a mechanism named *Task-agnostic Synonym Keyword to Sentence* pre-training. Next, a dual-view data augmentation approach is used to generate synthetic samples conditioned on the keywords in the input sample (Input View) and the input sample label (Output View), respectively. Keywords for the Input View are extracted using the unsupervised keyword extraction algorithm Rake [66]. Finally, a consistency filtering step is applied to only keep synthetic samples with consistent labeling.

**Fig. 1.**
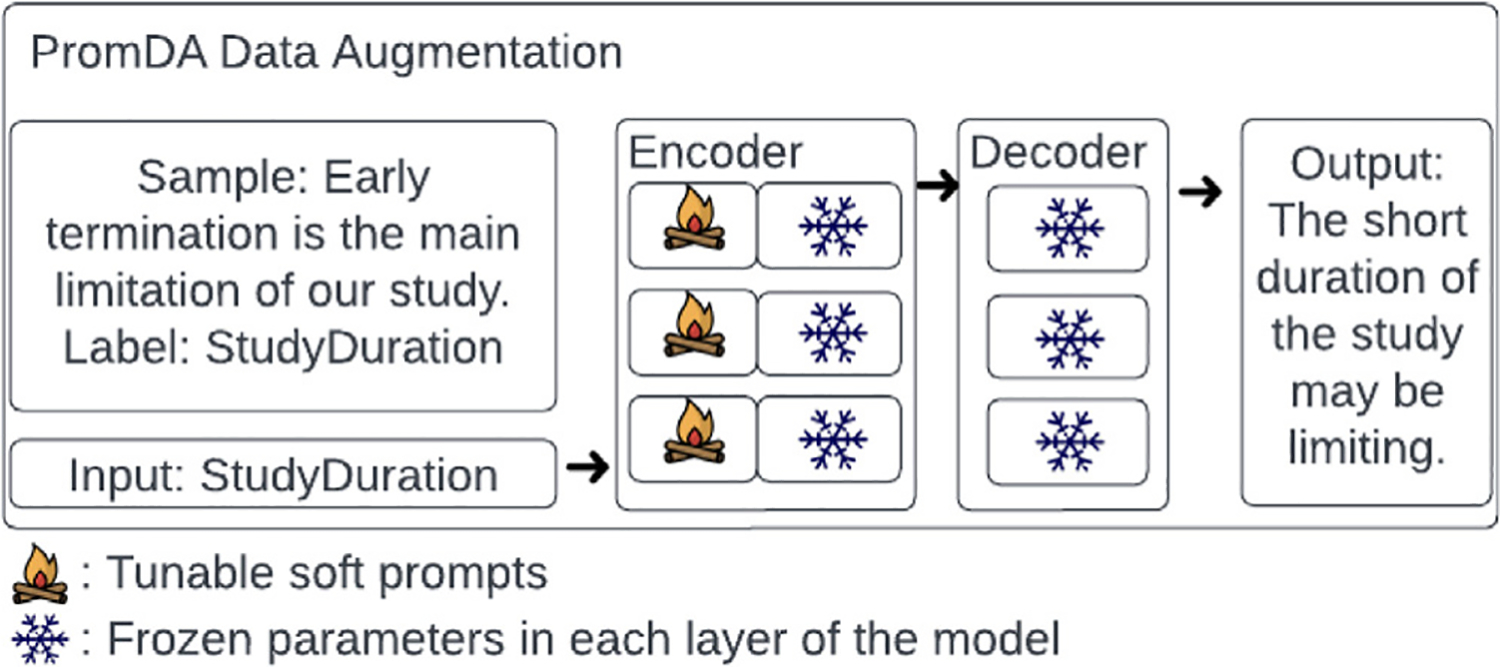
Overview of our soft-prompt based data augmentation (PromDA).

In this study, we use the soft prompt parameters pretrained on the *realnewslike* dataset [64]. We fine-tune the pretrained parameters using samples from the classes with fewer than 70 samples in our training set. Samples with multiple labels were excluded from data augmentation, since it was hard for the augmentation method to differentiate the features for each label. Funding label was excluded, because all sentences with this label were multi-label. In addition to original PromDA, we also experimented with augmentation with Input View and Output View only, because we observed that consistency filtering led to more examples for frequent labels and few examples for rare labels. We generated 10 synthetic examples for each original sentence. To augment the training set, we set the minimum number of samples for each class to 70. For classes with *n* samples in the training set (*n* < 70), we add 70 - *n* synthetic samples to the training set. The 70 – *n* samples are randomly selected from the synthesized sentences. Fig. 1 depicts the PromDA process.

#### 3.5.2. Oversampling

We also experimented with oversampling to address the issue of imbalanced data. Oversampling randomly duplicates samples from the minority classes to increase their representation in the training set. As in PromDA, we set the target size of each class to 70 and added 70 – *n* duplicate samples for classes with fewer than 70 samples, where *n* represents the number of original samples for the class.

#### 3.5.3. Easy Data Augmentation (EDA)

Another method we used for data augmentation was EDA [54], a simpler, rule-based method that synthesizes samples via simple modifications to the original sentence, including word order shuffle, random deletion/insertion, and synonym replacement. As in other methods, we set the target size of each class to 70.

#### 3.5.4. Rule-based identification

To address the Funding class, which was not augmented, we use a simple rule-based method, which labels a sentence as Funding, if stemmed tokens in the sentence contain the stems “*finance*” or “*fund*”.

### 3.6. Evaluation

We evaluated the SAL sentence classifier using precision, recall, and F_1_ score for the positive class and accuracy, in line with previous work. For SAL type categorization, we trained a baseline model by fine-tuning the PubMedBERT model on the manually annotated training set. We compared this model to those trained with augmented training sets (PromDA, PromDA – Input View, PromDA – OutputView, EDA). For comparison, we also trained the baseline model with the fine-grained labels. The model performances were measured using micro-precision, recall, and F_1_ score. To obtain reliable estimates of model performance, the models were trained and tested using five randomly initialized runs. We report the performance averages of these runs and the standard deviations. We use McNemar’s test [67] to determine whether the performance difference between the rule-based method and the PubMedBERT model for limitation sentence classification is statistically significant. To observe whether the data augmentation methods lead to statistically significant differences, we use Bhapkar test [68], a multi-class extension of McNemar’s test, and treat multi-label cases as additional classes.

### 3.7. Large-scale analysis of SALs

To describe SAL reporting at large scale, we used a set of 11,988 RCT articles (not included in our manually labeled dataset). This unlabeled dataset, curated from the PMC-OA subset in prior work [49], includes articles published from 2011 to 2020. We first applied the SAL sentence classifier to this dataset and extracted SAL sentences from the abstract, discussion- and limitation-related sections. We then applied the best-performing SAL type classification model to these sentences to predict limitation types.

## 4. Results

### 4.1. Dataset statistics

We annotated a total of 200 RCT articles, published between 2001 and 2022. 52 articles (26%) were published in general medical journals (e.g., BMJ), while the rest were published in specialty journals. 66 (33%) of the articles were published in journals with high-impact factors (defined as journal impact factor >= 10).

**Fig. 2.**
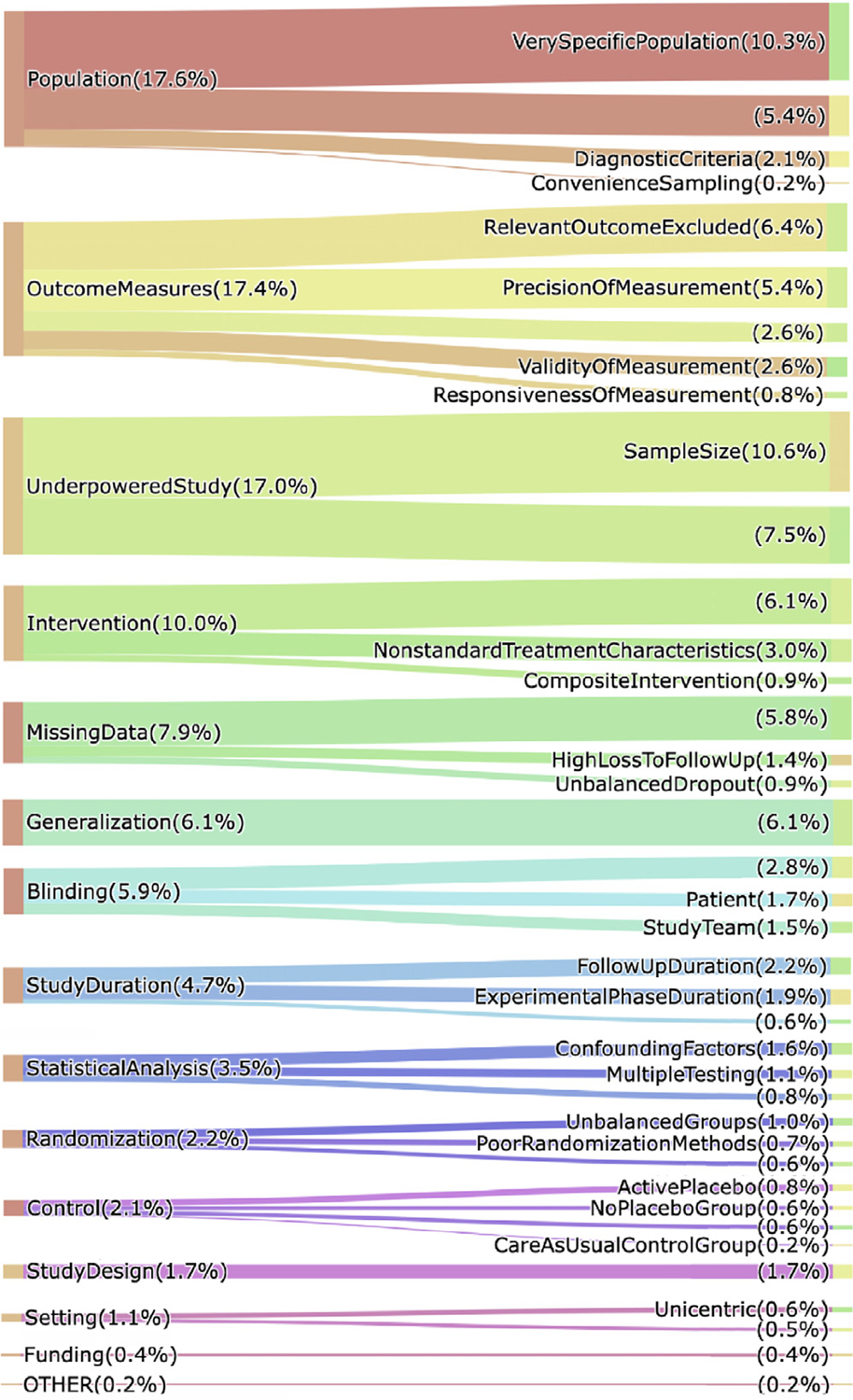
The sentence-level distribution of SAL types on the manually annotated dataset. Note that in some cases, the total number of fine-grained labels in a top-level category exceeds the total number for the top-level category, because the same sentence could be labeled with a top-level category as well as a fine-grained label belonging to the same top-level category (e.g., 10.6% + 7.5% > 17% for the UnderpoweredStudy category). The document-level distribution of SAL types on this dataset is provided in Appendix F.

A total of 1090 limitation types in 952 limitation sentences were annotated (1.15 and 5.45 limitation instances per sentence and per article, respectively). The top-level distribution and the count and percentage of sub-categories under each top-level category at the sentence level are presented in Fig. 2. They are also provided in Appendix A. Among the top-level categories, the most common limitation type was Population (192 out of 1090, 17.6%), closely followed by OutcomeMeasures (190) and UnderpoweredStudy (185). Limitations related to Funding (4) and Setting (12) were least reported. At fine-grained level, SampleSize (115), VerySpecificPopulation (112) and UnderpoweredStudy (82) were most discussed. The least mentioned fine-grained limitation types were ConvenienceSampling (2), CareAsUsualControlGroup (2) and Funding (4).

When we considered the unique limitation types reported in publications, we found that UnderpoweredStudy appears in 55% of the articles, closely followed by OutcomeMeasures (53.5%) and Population (52.5%) Appendix F.

Articles published in general medical journals had an average of 5.38 limitation sentences [95% CI: 5.26–5.51], and those published in specialty journals had an average of 4.56 sentences [95% CI: 4.50–4.62]. The average number of SAL types per general medical journal article was 3.75 [95% CI: 3.70–3.80] and that per specialty journal article was 3.45 [95% CI: 3.40–3.49].

Articles published in high-impact journals had an average of 5.23 limitation sentences [95% CI: 5.12–5.34], and those in lower-impact journals an average of 4.53 [95% CI: 4.46–4.60]. The average number of SAL types per high-impact journal article was 3.86 [95% CI: 3.79–3.94] and that per lower-impact journal article was 3.34 [95% CI: 3.30–3.37].

### 4.2. IAA

Sentence-level IAA (Krippendorff’s *α* with MASI) was 0.45 at the top level and 0.3 at the fine-grained level for 50 multiple-annotated articles over two annotation stages. In the second (last) stage of multiple annotation, they were 0.61 and 0.39, respectively, indicating some improvement in consistency over the first stage. Pairwise token-level agreement (Cohen’s *κ*) showed a range of 0.2–0.46 for the top-level categories and 0.11–0.3 for the fine-grained categories. There are no standard guidelines for interpreting Krippendorff’s *α*; however, the range of 0.6–0.8 is traditionally considered substantial agreement in the literature on agreement coefficients [69].

Given the low agreement at the token level and for fine-grained categories, we made the decision to focus on top-level categories and sentence-level classification for our NLP models. We note that all annotations were examined by at least two annotators, and verified for consistency by the annotator with the highest agreement with others (HK), which increases our confidence that the annotations can be used for training NLP models.

### 4.3. SAL sentence classification

The performance of the PubMedBERT-based SAL sentence classification model is reported in Table 2, along with the model performances from our previous work [27]. We obtained the best overall results with the PubMedBERT-based model proposed in this study (F1 score 0.821 vs. 0.806), mostly due to improvements in recall (0.907).

### 4.4. SAL type classification

Table 3 shows the performances of the SAL type classification models trained with and without data augmentation. For the baseline model (PubMedBERT fine-tuning), we present the model performances for both the top-level and fine-grained labels. Unsurprisingly, using a smaller set of labels (top-level) leads to better classification performance overall (about 18 absolute F_1_ points better, 0.671 vs. 0.494). We consider the model using the top-level categories our primary model.

Oversampling led to an overall degradation in model performance. While EDA improved recall, it also led to a drop in precision, F_1_ score remaining essentially the same. Although the original PromDA uses the most advanced technique, it led to a reduced performance (0.625 micro F_1_ score), which we attributed to the consistency filtering limiting the generation of examples for rare classes. When we only use the examples generated by PromDA (Output View) as additional training data, we obtain the best model performance (0.700 F_1_ score). We note that using only PromDA (Input View) also yields poorer results compared to the baseline. The effect of including data augmentation methods on model performance is statistically significant, except for oversampling (*p* < .05 for EDA and PromDA, and *p* < .001 for PromDA (Input View) and PromDA (Output View). We provide the results of the best-performing model for the top-level categories in Appendix Table D.1. We provide more details on data augmentation with PromDA in Appendix E.

### 4.5. Large-scale characterization of SALs in the RCT literature

**Fig. 3.**
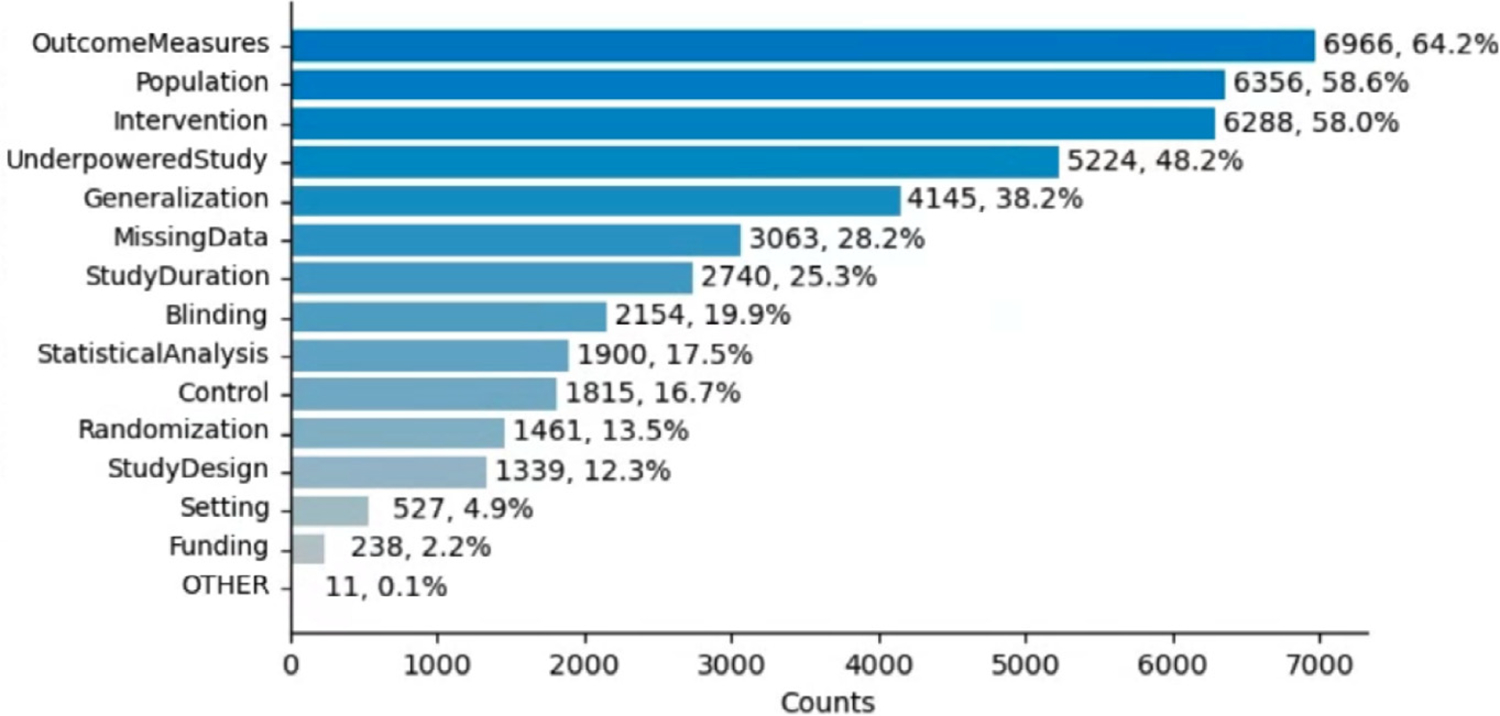
Document-level distribution of SAL types on the large-scale RCT dataset. *x*-axis shows the number of articles that contain a specific SAL type. The sentence-level distribution of SAL types on this dataset is provided in Appendix G.

From 11,988 RCT articles, our SAL sentence classifier identified 74,670 sentences out of 2,198,534 sentences as limitation sentences (4.23%). 10,843 RCT articles out of 11,988 had SAL sentences (90.4%, 6.2 sentences per article), which is close to the finding by Alvarez et al. [30] that 9% of RCT articles did not report SALs, while being higher than earlier estimates [7,11,27]. In Fig. 3, we present the SAL type distribution in this dataset, in terms of the number of RCT articles reporting the limitation, extracted by the PubMedBERT model trained with PromDA (Output View) data augmentation. OutcomeMeasures are most prevalent (64.2% of articles), followed by Population (58.6%), Intervention (58.0%) and UnderpoweredStudy (48.2%). These four types are also most common in the annotated dataset. The least common types in the large-scale RCT dataset were Setting (4.9%) and Funding (2.2%), also the least common in the annotated dataset.

## 5. Discussion

### 5.1. Limitation reporting

In our annotated dataset, Population, UnderpoweredStudy, and OutcomesMeasures were most common limitation types with similar prevalence, while Setting and Funding were the least common. This is largely consistent with the findings of Alvarez et al. [30], who found that UnderpoweredStudy was the most common limitation type, and Setting and Funding related limitations were the least reported. In the annotated dataset, the RCTs published in general medical journals and high-impact factor journals show higher limitation reporting compared to those in specialty journals and lower-impact journals. The finding for the general vs. specialty journal is consistent with the finding from ter Riet et al. [11].

In our large-scale analysis, we found that limitation types OutcomeMeasures, Intervention, in addition to UnderpoweredStudy and Population, were highly reported, while Funding and Setting remained the least reported types. About two-thirds of limitations reported seem to relate to the top four categories (approximately 62% in the annotated dataset and 71% in the larger corpus).

### 5.2. Data model and annotation

We started with the limitation type categorization in Alvarez et al. [70]. Because they focused on manual therapy RCTs, they included categories specific to that domain. Therefore, we created a modified categorization, which we believe is more generalizable. However, the fact that authors working in specific medical specialties may report limitations related to that domain suggests that the categorization may need to be extended for specialized domains. We consider this future work.

Annotation of SAL types was challenging. Our initial plan to annotate spans indicating fine-grained types yielded modest IAA due to the large number of fine-grained categories and the heterogeneity of limitation type expressions. Therefore, we mainly focused on a top-level sentence-level characterization for NLP. IAA at this level was comparable to agreement of experts in similar work [35,41,43]. We attempted to ensure high-quality annotations by having at least two annotators evaluate each article. The resulting dataset is relatively small, due to limited resources, and imbalanced, due to the nature of limitation reporting. However, we believe that it represents a good first step towards understanding the limitations of RCT studies, both explicit and implicit. We make the dataset publicly available to enable further studies in this area.

### 5.3. NLP models

Our results confirm PubMedBERT as a strong baseline system for supervised biomedical sentence classification and data augmentation as an effective strategy to address the data scarcity problem, common in biomedical NLP tasks. While the performance of the SAL sentence classifier seems reasonable for practical use, there is significant room for improvement for the SAL type classifier.

In this work, a prompt-based data augmentation method that uses a large language model (PromDA) helped the SAL type classifier achieve better performance. Interestingly, while Output View augmentation improved the performance, other mechanisms led to performance degradation. One potential explanation for the degradation due to Input View could be that the keyword extraction method, Rake, does not work well on biomedical text. Replacing this method with more recent methods, such as Yake [71] or KeyBERT [72], or using biomedical named entity recognition tools to identify important concepts could be a future direction. We also observed that consistency filtering diminished the possibility of generating examples of rare classes. The improved performance due to Output View augmentation suggests that label names are informative prompts for synthetic sentence generation using large language models. It seems plausible that SAL category definitions (Table 1) could additionally be leveraged for further performance improvements.

To better understand the generalizability of the best model, we assessed the accuracy of 250 predictions in the large-scale analysis set, which showed that 82% of sentences were correctly identified as the limitation sentences, and the SAL types in 77% of these limitation sentences were predicted correctly. These figures compare favorably to the precision of the sentence classifier and the SAL type classifier on the test sets (0.75 and 0.69, respectively) and suggest the generalizability of the model.

### 5.4. Error analysis

Table 4 shows three errors made by the best-performing model (PubMedBERT + PromDA-Output View). We leverage the saliency map created by integrated gradient algorithm [73] to gain insights into how the model focuses on sentence features. Specifically, the gradient integrals of the model’s output with respect to input features are calculated and presented by different colors; tokens assigned positive attention are highlighted in green and those assigned negative attention in red. Color intensity corresponds to the feature weight. In the first sentence, the features in the sentence, including “impossible”, “some”, and “between subjects”, were positively weighted, while tokens that seem more relevant for the gold label Blinding (“prevent”, “communication”) are negatively weighted, resulting in the incorrect prediction Intervention. In the second sentence, the model attends to the token “device” and less to the seemingly relevant tokens “consistency” and “standardization” for the gold label Generalization. In the third case, the token “participant” seems to have high weight, leading to the correct prediction Population, while the tokens relevant for the label StudyDuration (“short intervention period”) receive negative weight. This case also reveals the limited capability of our model in the multi-label setting.

### 5.5. Limitations of the study

Our study has limitations. First, the annotated corpus is small due to limited resources. We attempted to address this issue by using data augmentation for NLP. The inter-annotator agreement was modest (although similar to IAA in similar work, such as PICO classification [35, 41,43]). We also took additional steps to improve the data quality of the annotated corpus used for NLP. More specific RCT domain expertise, more detailed annotation instructions, and more extensive discussions of annotations could have further improved data quality. The data model extended a data-driven characterization based on manual therapy RCTs [30]. While we believe our characterization is more broadly applicable, a more theoretically sound characterization based on causal inference literature [74] could have further improved generalizability. At the same time, our model may lack more fine-grained categories that might be more practically relevant to specialized domains. Our large-scale analysis is limited by the fact that we only considered a subset available from PMC-OA and the underlying model is imperfect. Lastly, we note that SALs may differ from the “real” limitations of a study as perceived by an RCT methodologist.

## 6. Conclusions

We presented the first NLP work on labeling and automatic identification of SAL types reported in RCT articles (and scientific literature, more broadly). We also improved a previously reported SAL sentence classification model. While the latter model performs well and has been incorporated into a COVID-19 preprint screening pipeline [23,24], there is significant room for improving the performance of the SAL type model, which we will explore in future work. We also reported a large-scale analysis of RCT literature based on our model, which is the first of its kind.

In future work, we will focus on improving model performance via zero-shot and few-shot learning approaches that leverage generative large language models. With sufficient accuracy, such models can be incorporated into the peer review workflows to better surface the potential weaknesses of a study. They can also facilitate large-scale studies on *meta-research* [75]. Our longer term goal is to develop methods to assess studies not only for limitations that are self-acknowledged but also those that are implicit.

## Supplementary Material

Annotation guidelines

## Figures and Tables

**Table 1:** Types and descriptions of self-acknowledged limitation types.

Top-level	Fine-grained categories	Description
StudyDesign		Limitations that have to do with the specific trial design used (e.g., crossover, factorial, cluster, etc.).
Population		Limitations that have to do with the selection of subjects who participated in the trial.
	DiagnosticCriteria	Lack of standardized diagnostic criteria for including participants.
	VerySpecificPopulation	Inclusion criteria considered too restricted (e.g., single gender, athletes only, education level, or race).
	ConvenienceSampling	The sampling method was linked to specific study needs. Subjects were selected because they were convenient sources of data for the study.
Setting		Limitations related to where the study takes place.
	Unicentric	Study was conducted recruiting participants from a single center.
Intervention		Limitations that have to do with the active intervention treatment used.
	CompositeIntervention	It was not possible to know the net effect of every component in multimodal treatments.
	NonStandardTreatmentCharacteristics	The specific parameters for the intervention were not standardized (e.g., dosage, mode of administration).
Control		Limitations that have to do with the control intervention placebo.
	NoPlaceboGroup	No control intervention is included.
	ActivePlacebo	An active intervention (non-inert) was selected as control.
	CareAsUsualControlGroup	Due to the non-standardization of CAU, it is uncertain what it is that the experimental group is being compared to. In these cases, the control group treatment will sometimes be mentioned as “care as usual”.
Outcome Measures		Limitations related to the outcomes used and how they are measured.
	RelevantOutcomeExcluded	Some relevant data that would potentially provide interesting findings were not collected during the study.
	PrecisionOfMeasurement	Lack of or low precision of outcome measures. This refers to a limitation due to random errors that might have been introduced in measurement.
	ValidityOfMeasurement	The selected assessment instrument was not originally validated for the specific population or problem studied. This indicates that the outcome measurement may not correctly measure the concept that is the target of the measurement (systematic error, as opposed to random error).
	ResponsivenessOfMeasurement	Outcome measures were not sensitive enough to detect subtle changes (e.g., use of ordinal scales). Responsiveness is defined as the ability of an instrument to accurately detect change when it has occurred.
MissingData		Some data were not collected for some study participants. This indicates that some planned follow-up measurements, whether outcomes or co-variables (confounders) were not collected, regardless of the reasons for that missingness.
	HighLossToFollowUp	Many participants stopped participating before the planned duration of follow-up.
	UnbalancedDropout	Characteristics of dropped out patients differed between groups (‘informative drop-out’). For example, relatively healthy patients dropped out from the experimental group, whereas patients in relatively poor health dropped out from the control group.
Underpowered Study		Inability to detect differences between groups due to sample size or insufficient number of outcome events.
	SampleSize	Limitations related to the insufficient number of patients participating in the trial. This may be a result of recruitment difficulties.
Randomization		Limitations that have to do with the randomization of patients into different trial arms.
	UnbalancedGroups	After randomization, there were large differences between the groups with respect to (mean values of) prognostically important factors (confounders). This is problematic because the response to interventions may be due to these confounders, rather than the interventions.
	PoorRandomizationMethods	Randomization methods used (e.g., for sequence generation, restriction stratification, concealment) were not optimal. This also includes a lack of such methods, e.g., that allocation was not concealed (i.e., once a patient is assigned, the next assignment is predictable).
Blinding		Limitations related to how the study participants and personnel were blinded to the study groups.
	Patient	Patients were not blinded with respect to the study groups.
	StudyTeam	Some people in the study team (investigators, care providers, outcome assessors, statisticians etc.) are not blinded.
StudyDuration		Limitations that have to do with the length of the study. It could be the experimental phase or the follow-up.
	ExperimentPhaseDuration	The intervention phase is too short. It could be due to early stopping.
	FollowUpDuration	Only short term effects were evaluated. Long term (adverse) effects of the interventions were not considered.
Statistical Analysis		Limitations regarding the methods used for statistical analysis, indicating that the techniques used may not have been appropriate or were suboptimal.
	MultipleTesting	Simultaneous testing of more than one hypothesis.
	ConfoundingFactors	Findings were not adjusted for covariates.
Funding		The limited or lack of funding affected the study progress or completion.
Generalization		The study results were compromised and may not generalize due to type of setting, specific population, intervention, and measurement instruments.
Other		Catch-all category for all limitation types that do not neatly fit in any of these categories.

**Table 2:** Performance comparison of limitation sentence classifiers. The performance difference between the PubMedBERT model and the rule-based method is statistically significant (McNemar’s test: *p* < .001). We were unable to calculate the statistical significance of the performance difference with the SVM models, because their predictions were unavailable.

Method	Precision	Recall	F_1_	Accuracy
Rule-based method^a^	0.758	0.848	0.800	0.915
SVM^a^	0.766	0.693	0.728	0.896
SVM + self-training^a^	0.778	0.835	0.806	0.919
PubMedBERT (this work)	0.751	0.907	0.821	0.929

a denotes the results from previous work [27].

**Table 3:** Micro-precision, recall and F_1_ scores for SAL type classification models. The average and the standard deviation over 5 randomly initialized runs are reported. The statistical significance of the performance difference between vanilla PubMedBERT model and models that use data augmentation are calculated using Bhapkar test and are shown with asterisks (*: *p* < .05, **: *p* < .001). The performance difference with the oversampling model is not statistically significant (*p* = .055). Macro-level scores are provided in Appendix C.SD: standard deviation

Prediction Level	Method	Precision ± SD	Recall ± SD	F_1_ ± SD
	PubMedBERT	0.680 ± 0.024	0.666 ± 0.011	0.673 ± 0.010
	+ Oversampling	0.659 ± 0.024	0.646 ± 0.035	0.652 ± 0.028
Top-level	+ EDA*	0.664 ± 0.015	0.679 ± 0.003	0.671 ± 0.008
	+ PromDA Original*	0.631 ± 0.043	0.619 ± 0.039	0.625 ± 0.039
	+ PromDA (Input View)**	0.643 ± 0.020	0.633 ± 0.027	0.638 ± 0.022
	+ PromDA (Output View)**	0.690 ± 0.011	0.711 ± 0.015	0.700 ± 0.007
Fine-grained	PubMedBERT	0.488 ± 0.019	0.500 ± 0.021	0.494 ± 0.017

**Table 4:** Examples of errors made by the best-performing PubMedBERT PromDA-Output View model. Word Importance column indicates how the classifier focuses on the sentence features. If a feature is assigned positive attention, it is highlighted in green. Conversely, a feature is assigned negative attention and highlighted in red indicates bias might be introduced. The intensity of the color corresponds to the feature’s weight.

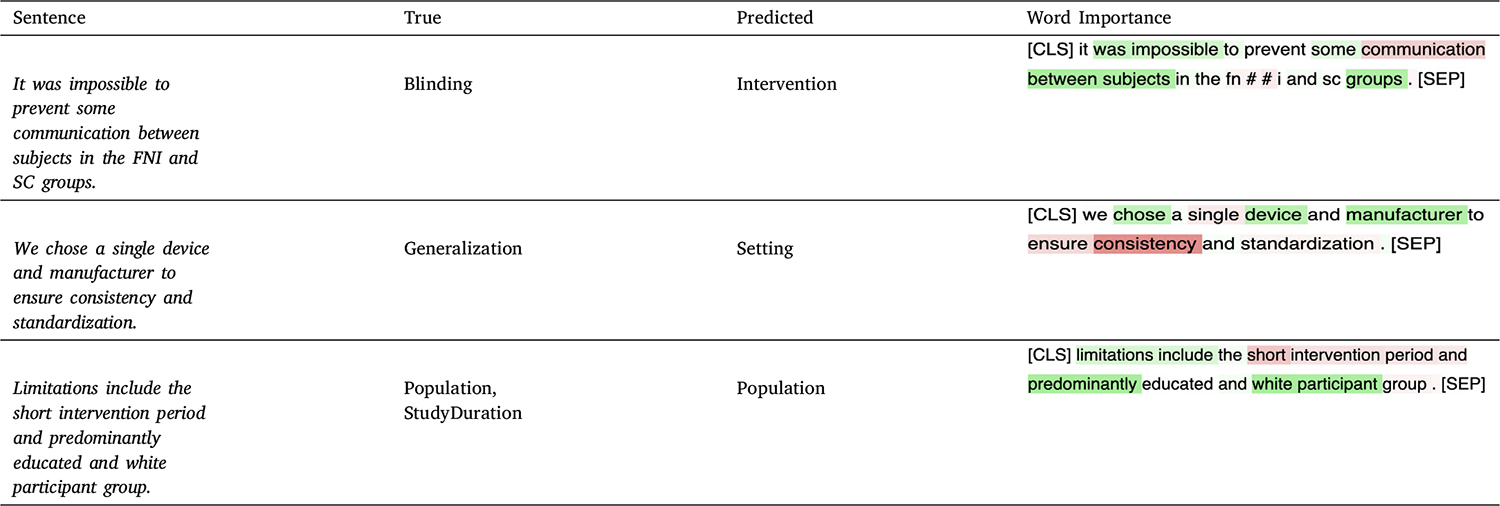

**Fig. E.4.**
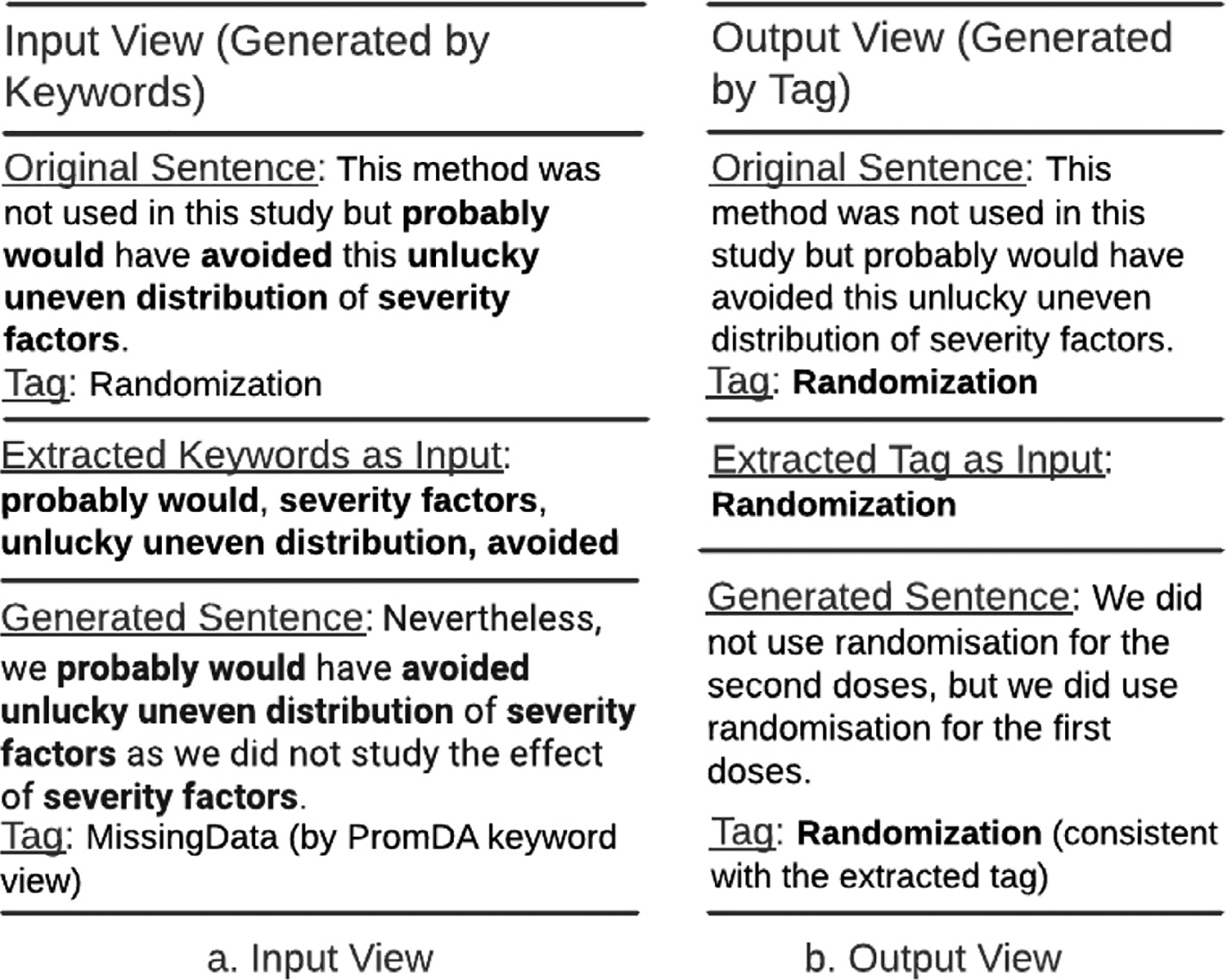
A sample generated by output-view.

**Fig. F.5.**
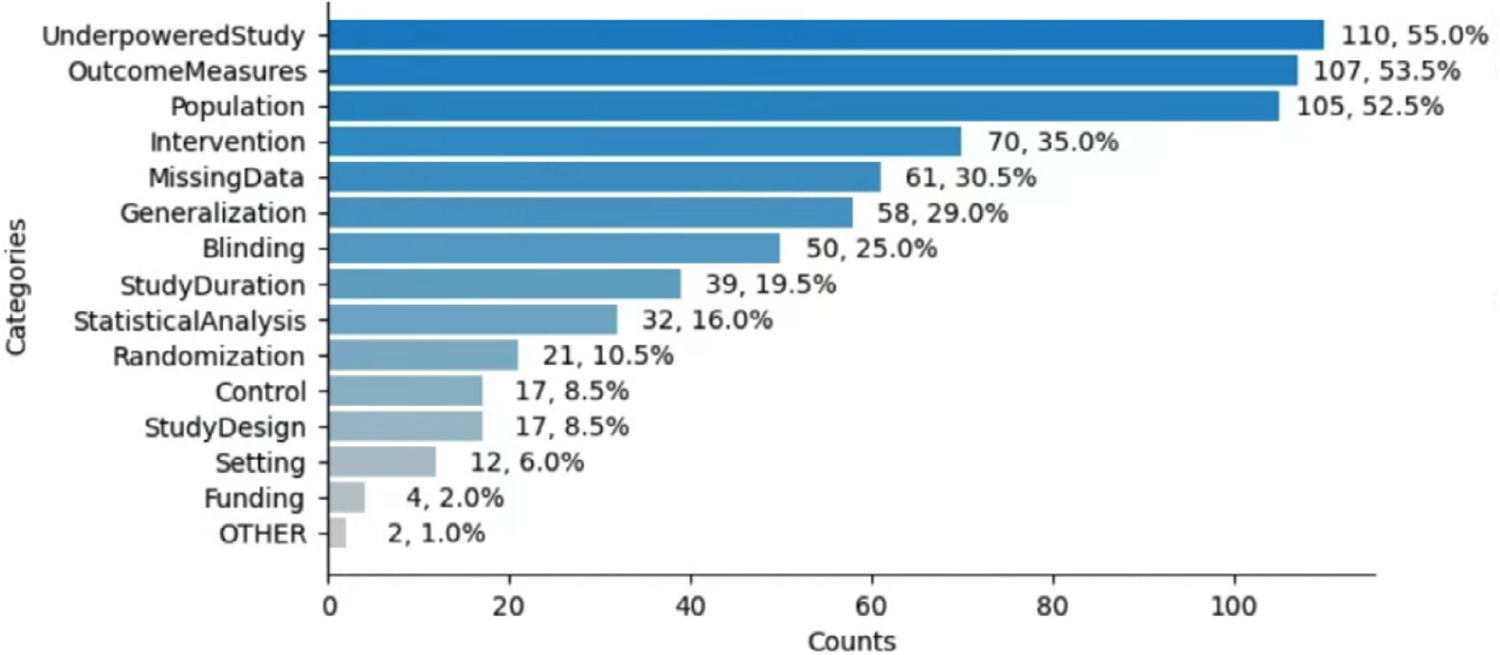
Document-level distribution of SAL types on the manually annotated dataset. The *x*-axis shows the number of documents that contain a specific SAL type.

**Fig. G.6.**
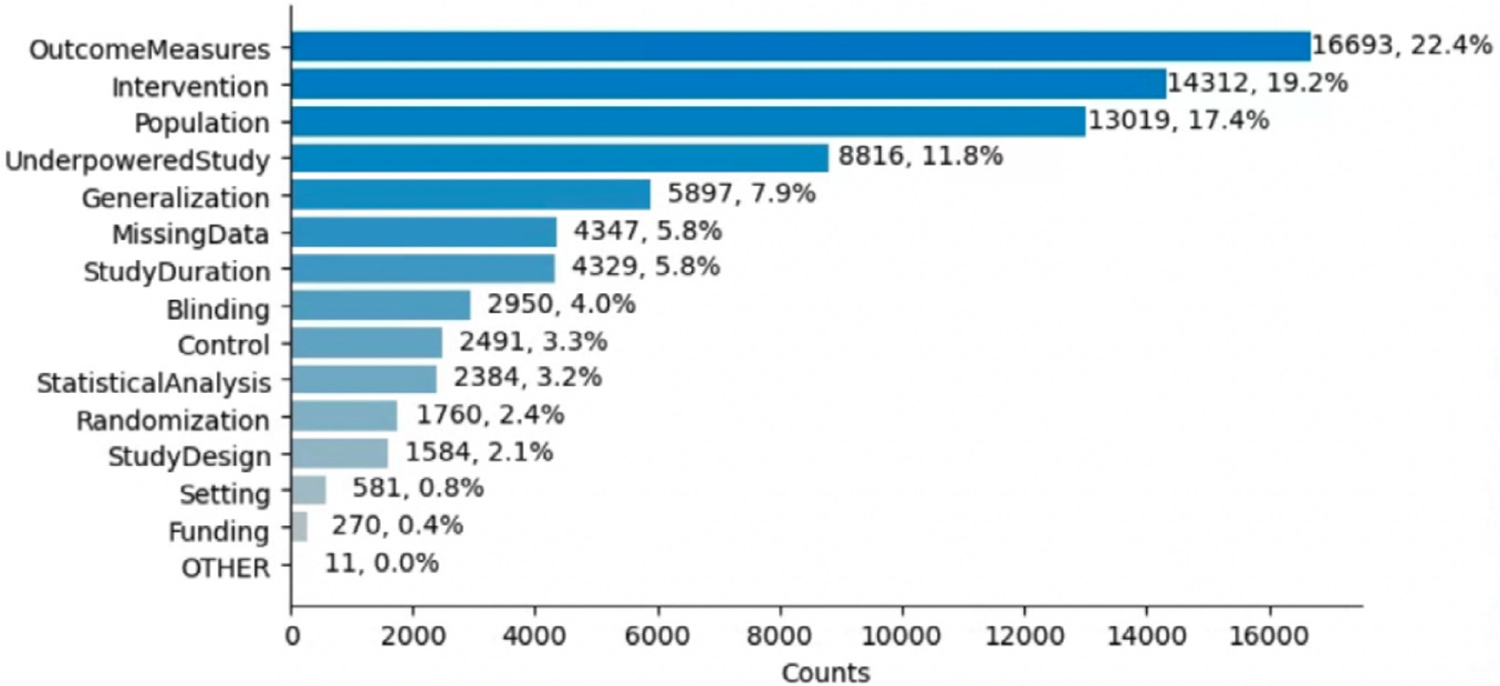
Sentence-level distribution of SAL types on the large-scale RCT dataset. The *x*-axis shows the total number of sentences with the SAL types in the dataset.

## Data Availability

The limitation type dataset, the PubMedBERT models, and the source code are available at https://github.com/MengfeiLan/SAL_Type_Classification.

## Appendix A. Limitation type categories with example sentences

See Table A.1.

**Table A.1:** Limitation type categories with example sentences in the annotated dataset. Spans relevant to the categorization are highlighted in bold.

Top-level	Fine-grained categories	Description
StudyDesign (19)	(19)	*One study limitation is* ***the use of a non-randomized control group***.
Population (192)	(59)	*The unequal distribution of gender in the study sample may also have influenced the results*.
	DiagnosticCriteria (23)	*We defined* ***no exclusion criteria related to severity of background illness or sepsis***.
	VerySpecificPopulation (112)	*While the sample was randomly selected*, ***selection bias is possible*** *due to the 64% response rate*.
	ConvenienceSampling (2)	*We did not recruit a representative sample; thus study results cannot be used to draw conclusions about hypothesis testing or population-level dynamics*.
Setting (12)	(5)	*Educational classes were an important activity in this trial and* ***the uptake of information could have been better in larger schools***.
	Unicentric(7)	*Finally the characteristic of being* ***a monocentric trial*** *can be considered both a limitation but also an advantage due to reduction of variability of care*.
Intervention (109)	(67)	*Finally, while participant retention and compliance with outcomes protocols was high (loss to follow-up was 2%)*, ***adherence to TC training*** *was lower than expected*.
	CompositeIntervention (10)	*We chose not to have an intervention group receiving BMD feedback alone without any other educational intervention*.
	NonStandardTreatmentCharacteristics (33)	*One possible reason for the lack of effect is that* ***families did not comply with the intervention***.
Control (23)	(6)	*The absence of another control comparator makes it difficult to attribute the beneficial effects solely to the intervention*.
	NoPlaceboGroup (6)	*To prevent this bias*, ***a placebo treatment group with patients receiving an equal amount of therapist attention would be required***.
	ActivePlacebo (9)	*First, it is known that* ***the controlled substance propofol also has protective characteristics***.
	CareAsUsualControlGroup (2)	*Our choice of* ***a usual care control*** *followed the overarching practical goal of our study—to evaluate the potential benefits to osteopenic women of adding TC to usual care*.
Outcome Measures (190)	(28)	*We are also limited by* ***our exclusive focus on intra-individual factors as moderators***.
	RelevantOurcomeExcluded (66)	*Genotyping, which was not practical in our study setting, might have aided the interpretation of our findings*.
	PrecisionOfMeasurement (59)	*However*, ***self-reported outcome by patients is necessarily subjective*** *and affected by many things besides knowledge of treatment allocation*.
	ValidityOfMeasurement (28)	*The difficulty of capturing all dimensions of physical activity by questionnaire is well-known*[38,39], *particularly non-leisure activities in women*[40].
	ResponsivenessOfMeasurement (9)	*There are several possible explanations for this, including the possibility that* ***our measures were not sensitive enough over this time period***.
MissingData (86)	(63)	*Furthermore*, ***we do not have complete information about adherence to study medication***.
	HighLossToFollowUp (15)	*Our loss to follow-up for our functional secondary outcomes measured at ICU* ***and hospital discharge was also significant*** *as patients were often unable to participate in the assessments*.
	UnbalancedDropout (10)	*This impression of practical difficulty is reinforced by* ***the significantly higher proportion of participants that withdrew from the trial in the intensive arm***.
Underpowered Study (185)	(82)	*The subgroup analysis is therefore confronted with an even poorer lack of power*.
	SampleSize (115)	*Even when the remaining girls complete the study, the number evaluable at* ***final height will still only be 92***.
Randomization (24)	(6)	*Another limitation is that* ***we performed simple randomization*** *instead of block randomization*.
	UnbalancedGroups (11)	*This method was not used in this study but probably would have avoided* ***this unlucky uneven distribution of severity factors***.
	PoorRandomizationMethods (8)	*Although* ***open allocation*** *was an unavoidable limitation of the monitoring randomization and was not undertaken for the ART-strategy randomization, the endpoint review committee adjudicated endpoints masked to randomization*.
Blinding (64)	(31)	*Therefore, it is not clear why bias (or* ***lack of blinding****) would not have similarly led to more crossovers from the RF to the conventional needle*.
	Patient (19)	*First*, ***subjects were not blinded to their intervention group***.
	StudyTeam (16)	*One major limitation was that* ***the first author conducted all aspects of the trail including provision of care to all study participants***.
StatisticalAnalysis (38)	(9)	*Furthermore, due to the exploratory nature of the feasibility study*, ***no multiple outcomes or sample size calculation were performed***.
	MultipleTesting (12)	*Finally, we conducted a number of statistical tests, raising* ***the potential concern of alpha inflation***.
	ConfoundingFactors (17)	*Differences in age, stress, depression, and other factors can potentially influence* ***success in managing weight, and may have confounded results***.
StudyDuration (51)	(6)	*The short duration precluded the use of change in HbA levels as an efficacy end-point*.
	ExperimentPhaseDuration (21)	*Study duration is another potential study limitation given the long half-life of OKZ (31days)*.
	FollowUpDuration (24)	*Longer-term follow-up is needed to confirm any lasting positive effects on BMD from ongoing calcium supplement use*.
Funding (4)	(4)	*The trial was stopped after achieving 86% of target recruitment owing to time and* ***financial limitations***.
Generalization (66)	(66)	*As such*, ***our sample might not be generalizable to all those who experience grade 1 and 2 ankle injuries***.
Other (2)	(2)	*This pilot trial involved co-funding and participation by the device manufacturer*.

## Appendix B. Experimental settings

To fine-tune the PubMedBERT model for limitation sentence classification, we set the maximum sentence length to 512 tokens and tuned all the hyperparameters on the development set. The following hyperparameters were used: AdamW optimizer, batch size: 4, learning rate: 3e-5, and epochs: 10.

We split the manually annotated dataset (200 articles) to train/dev/test sets as 120/40/40. To tune the PubMedBERT-based models SAL classification, we also set the maximum sentence length to 512 tokens and tuned the hyperparameters on the development set. The following hyperparameters are used for the reported models: AdamW optimizer, batch size: 4, learning rate: 1e-5, epochs: 20. All experiments were conducted on a Tesla T4 GPU.

## Appendix C. SAL type classification results (macro-averaging)

See Table C.1.

**Table C.1:** Macro-precision, recall and F_1_ score of SAL type classification results. The average and the standard deviation over 5 randomly initialized runs are reported. SD: standard deviation.

Prediction level	Method	Precision ± SD	Recall ± SD	F_1_ ± SD
Top-level	PubMedBERT	0.552 ± 0.033	0.514 ± 0.039	0.504 ± 0.026
	+ Oversampling	0.535 ± 0.054	0.517 ± 0.088	0.490 ± 0.066
	+ EDA	0.577 ± 0.047	0.577 ± 0.024	0.534 ± 0.017
	+ PromDA Original	0.508 ± 0.039	0.495 ± 0.651	0.453 ± 0.038
	+ PromDA (Input View)	0.520 ± 0.033	0.522 ± 0.023	0.482 ± 0.029
	+ PromDA (Output View)	0.611 ± 0.011	0.632 ± 0.035	0.583 ± 0.018
Fine-grained	PubMedBERT	0.301 ± 0.021	0.288 ± 0.032	0.266 ± 0.026

## Appendix D. Detailed results for the best-performing model (PromDA – Output View)

See Table D.1.

**Table D.1:** Performance of the best model (PromDA – Output View) for the top-level categories. The results are from the best-performing run among the five runs.

Category (Top-level)	Precision	Recall	F_1_
StudyDesign	0.500	0.286	0.364
Population	0.700	0.700	0.700
Setting	0.500	0.500	0.500
Intervention	0.364	0.727	0.485
Control	0.250	1.000	0.400
OutcomeMeasures	0.726	0.787	0.755
MissingData	0.556	0.588	0.571
UnderpoweredStudy	1.000	0.800	0.889
Randomization	0.500	1.000	0.667
Blinding	0.842	0.842	0.842
StudyDuration	0.900	0.643	0.750
StatisticalAnalysis	0.800	0.364	0.500
Funding^a^	1.0	1.0	1.0
Generalization	0.769	0.625	0.690
Other	0.0	0.0	0.0
Micro-average	0.705	0.699	0.702
Macro-average	0.627	0.657	0.608

a Results for Funding are obtained using a rule-based method.

## Appendix E. Data augmentation with PromDA

To illustrate the difference between the generated sentences by PromDA Input-view and Output-view, we present two samples in Fig. E.4. The example in Fig. E.4a shows the keywords “probably would”, “severity factors”, “unlucky uneven distribution”, and “avoided” are extracted and fed as input to generate text. The generated sentence containing the keywords is assigned with the label Missing Data by the Input View method. However, the label assigned to the generated sentence does not seem appropriate. Another sample presented in Fig. E.4b is generated by the Output View. The tag Randomization is fed as input to the model. Although the output sentence contains much novel information not seen in the original sentence, it maintains semantic coherence and is suitable to maintain the original sentence label as Randomization.

Table E.1 shows further examples generated by PromDA-Output View. In the first case, data augmentation leads to a sentence that contains a near-synonym of the original text (*short duration* instead of *early termination*). In the second sample, the augmentation method paraphrases and extends part of the original sentence *we did not obtain prospective data for cardiovascular comorbidity and causes of death* as *we also had limited data on comorbidity, mortality and mortality (11%)*, while retaining the important limitation type-related information. In the third case, the synthesized sentence also keeps the important limitation-related keyword *monocentric*, although the sentence is less than semantically coherent due to the clause *In the absence of a single center trial*.

**Table E.1:** PromDA Output-view Augmentation.

Original Sentence	PromDA Augmentation	Label
*Early termination is the main limitation of our study*.	The short duration of the study may be limiting.	StudyDuration
*Second, because of the nature of pragmatic trials, we did not obtain prospective data for cardiovascular comorbidity and causes of deaths, including results of post-mortem*.	We also had limited data on comorbidity, mortality and mortality (11%).	MissingData
*Finally the characteristic of being a monocentric trial can be considered both a limitation but also an advantage due to reduction of variability of care*.	In the absence of a single center trial, a monocentric trial approach may be more suited for patients with anaemia with a larger pulmonary embolism.	Setting

## Appendix F. Document-level distribution of SAL types on the manually annotated dataset

See Fig. F.5.

## Appendix G. Distribution of SAL types on the large-scale RCT dataset

See Fig. G.6.

## Appendix H. Supplementary data

Supplementary material related to this article can be found online at https://doi.org/10.1016/j.jbi.2024.104628.
