# Supplementary material for: Automatic categorization of self-acknowledged limitations in randomized controlled trial publications
Source: J Biomed Inform. Author manuscript; Available in PMC 2025 Feb 9. (PMC11807350; doi:10.1016/j.jbi.2024.104628)
Supplement: Annotation guidelines [file NIHMS2053914-supplement-Annotation_guidelines.pdf]

Annotation Guidelines

In this project, we annotate the types of limitations that are self-acknowledged by the authors in randomized controlled trial (RCT) publications. This is a continuation of the earlier project on identifying self-acknowledged limitation sentences in clinical publications[1].

In the first step of this annotation project, you will be provided RCT articles for which at least one sentence has been previously annotated as being a limitation sentence. The task is to identify the specific type of limitations discussed in these sentences and annotate the precise spans of text that support these limitation types.

The limitation type categorization has been adapted from Alvarez et al.[2] and contains 16 main categories and subcategories for each in this classification (shown at the end of this document). We provide the ability to annotate at both levels to see whether each category can be identified reliably. An OTHER category is provided as a catch-all category for all limitation types that do not neatly fit in any of these categories.

The annotation will be performed using the *brat* annotation tool. The URL for annotation is: <URL>. Each annotator has her/his own folder and should only annotate in that folder. Each document in the annotation set corresponds to a RCT publication. The PubMed Central ID of the publication is in the document title[3]. The first words of the limitation sentences in the publication are marked with the label Limitation (you may need to scroll down, since limitation sentences are mainly in Discussion sections). These are the main sentences to focus on in the project. An example screenshot is below.

---

[1] Kilicoglu H, Rosemblat G, Malički M, ter Riet G. Automatic recognition of self-acknowledged limitations in clinical research literature. *Journal of the American Medical Informatics Association*. 2018;25(7):855-61.

[2] Alvarez G, Núñez-Cortés R, Solà I, Sitjà-Rabert M, Fort-Vanmeerhaeghe A, Fernández C, Bonfill X, Urrútia G. Sample size, study length, and inadequate controls were the most common self-acknowledged limitations in manual therapy trials: A methodological review. *Journal of Clinical Epidemiology*. 2021;130:96-106.

[3] Brat annotation tool removes all the formatting, so these IDs might be useful in locating the articles on PubMed Central to see its structure more clearly.

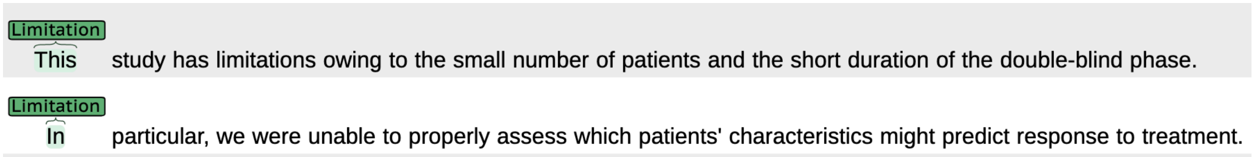

The first sentence above reports a limitation of sample size. The span that indicates this type can be "the small number of patients". To annotate this, select the phrase "the small number of patients" and select Sample Size from the drop-down category list that pops up (UnderpoweredStudy->SampleSize). After this annotation is added, the sentence looks as follows.

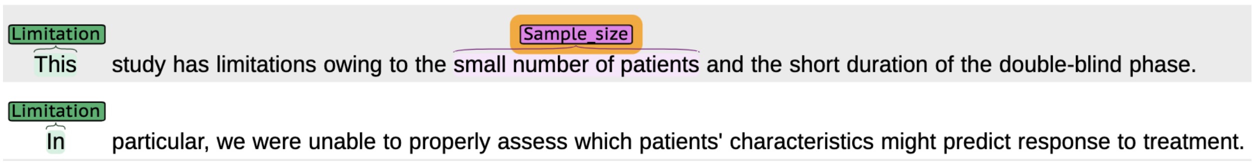

Guidelines/caveats:

- Annotate the selected phrase with the most specific limitation type in the categorization that you can identify as long as this type is explicit in the text.
- In annotating phrases, we follow a minimality principle to ensure consistent annotations. In other words, we annotate the shortest span that indicates the relevant category. Generally speaking, an annotated span should be a full clause/phrase and should make sense in isolation.
    - Ask the question "what is the limitation of the study?". Mark the clause that can fill the blanks in "The limitation of the study is _____" or "The limitation of the study is that _______".
        - e.g., The limitation of the study is "the small sample size".
        - The limitation of the study is that "patients allocated to tranexamic acid may have had a more stable circulation"

- Exclude consequences of the limitation that are discussed, if possible. Avoid labeling inferences/interpretation made by the authors based on the limitation reported.
  - e.g., in the sentence "the lack of a no treatment arm in this study means we cannot compare these cure rates with the natural resolution rate", limit your annotation to "the lack of a no treatment arm", as the second part of the sentence describes a consequence of the limitation.
- Relatedly, do not over-interpret what is said in the article and try to stick to what the authors of the article describe as their shortcomings/limitations. Note that some of these "reported" limitations may not accurately reflect the "actual" limitations of the study, but that is okay, since we are interested in "reported" limitations. Actual limitations may be very hard to understand from the papers only.
  - e.g., in the sentence "The absence of masking is most relevant for the assessment of the events within 7 days." annotate "the absence of masking" as Blinding. Although it may be possible to infer a more specific type like Patient blinding, this is not explicit.

- When the same sentence discusses multiple kinds of limitations, annotate them separately.
  - This small sample size [Sample Size], along with the heterogeneity of the study population [Diagnostic Criteria], resulted in high variability, quantified by the PBO groups.
    - Note that we do not annotate "high variability" as a limitation, since it is a consequence, as indicated above.
- Only annotate in the sentences that are marked as Limitation sentences. We do this so that the sentences annotated can be more consistent among annotators. We make sure that all sentences marked as Limitation sentences indeed report limitations.

- Use OTHER sparingly. If you cannot identify a subcategory that is relevant, but the limitation fits in with the higher level category (e.g., Population), annotate the higher level category, rather than OTHER.

Limitation Categories with examples:

- **Study Design:** Limitations that have to do with the specific trial design used (e.g., crossover, factorial, cluster, etc.).
    - *However, the crossover design and the relatively short, 4-week, treatment periods are limitations with respect to drawing conclusions from the ACQ-7 score analysis.*
- **Population:** These are limitations that have to do with the selection of subjects who participated in the trial.
    - Diagnostic criteria: Lack of standardized diagnostic criteria for including participants. An example of this is when the trial involves too heterogeneous a population (i.e., the eligibility criteria is somewhat loose).
        - *The definition of an active DU used in this trial may not match criteria used in other centres.*
    - Very specific population: Inclusion criteria considered too restricted (e.g., single gender, athletes only, education level, or race). In some sense, this is the opposite of the Diagnostic criteria category. The trial population is so narrow that, as a result, its findings may not generalize to other relevant populations.
        - *Cases of grade 3 and grade 4 envenomation were not included in the present study, as required by the ethics committee.*
    - Convenience sampling: The sampling method was linked to specific study needs. Subjects were selected because they were convenient sources of data for the study.
- **Type of setting:** Limitations related to where the study takes place.

- *The heterogeneous population characteristics, geographical differences in recruitment [Setting] (most patients were from Vietnam but there were no significant differences between Vietnam and other sites), and the variety of infecting viruses in our trial reflect the clinical circumstances in South East Asia…*
- Unicentric: Study was conducted recruiting participants from a single center. The common consequence of this is that it may not generalize.

- **Intervention:** Limitations that have to do with the active intervention/treatment used. Active intervention can be a drug, a procedure, exercise, etc. that is being evaluated in the trial.
  - Composite intervention: It was not possible to know the net effect of every component in multimodal treatments. The intervention has multiple parts to it, and disentangling the effects of individual components is difficult.
  - Non-standard treatment characteristics: The specific parameters for the intervention were not standardized (e.g., dosage, mode of administration)
    - *To some extent, the variability in the dose administered makes determination of metformin's efficacy more difficult.*
- **Control:** Limitations that have to do with the control intervention/placebo. This is what is compared to the active intervention in the trial.
  - No placebo/sham group: No control intervention is included.
  - Active placebo/sham: An active intervention (non-inert) was selected as control. The placebo is expected to be a non-drug (passive), but in the trial, an actual drug or intervention was used as control.
    - *Hence it was included in both the arms of the trial and no placebo group was included*
  - Care-as-usual (CAU) control group: due to non-standardization of CAU, it is uncertain what it is that the experimental group is being compared to. In these cases, the control group

treatment will sometimes be mentioned as "care as usual." This is a limitation because CAU is not specific and also may not qualify as an appropriate control.

- **Outcome measures:** Limitations related to the outcomes used and how they are measured.
  - Relevant outcome excluded: Some relevant data that would potentially provide interesting findings were not collected during the study. Note that uncollected data is different from missing data for the outcomes that were included in the study (which is now a top-level category, see below) and points at a more conceptual limitation of the study.
    - *Genotyping, which was not practical in our study setting, might have aided the interpretation of our findings.* **Note:** Here, genotyping was not used at all.
  - Precision of measurement: Lack of or low precision of outcome measures. This refers to a limitation due to random errors that might have been introduced in measurement
    - *The determination of the point at which symptoms disappeared was thus subjective, but we think that this was a clinically relevant and pragmatic way of judging the effects of treatment in a trial carried out in challenging circumstances with restricted resources in a rural setting in India.*
  - Validity of measurement: The selected assessment instrument was not originally validated for the specific population or problem studied. This indicates that the outcome measurement may not correctly measure the concept that is the target of the measurement (systematic error, as opposed to random error (see precision, above).
    - *Although the radiographic endpoints used are widely reported and validated, they remain surrogate measures that rely on visual representation and interpretation of joint damage and can be subject to error and bias.*
  - Responsiveness of measurement: Outcome measures were not sensitive enough to detect subtle changes (e.g., use of ordinal scales). Responsiveness is defined as the ability of an instrument to accurately detect change when it has occurred.

        - *There are several possible explanations for this, including the possibility that our measures were not sensitive enough over this time period.*

- **Missing data:** Some data were not collected for some study participants. This indicates that some planned follow-up measurements, whether outcomes or co-variables (confounders) were not collected, regardless of the reasons for that missingness. If missing data occur in co-variables (i.e. non-outcome variables or confounding variables) the potential to statistically adjust or correct the study findings during data-analysis will be reduced.
    - *In each year of post-trial follow-up, about a fifth of the surviving participants did not return their postal questionnaire.*
    - High drop-out rate: Many participants stopped participating before the planned duration of follow-up. Often authors will use the term loss to follow-up, or discontinuation, which are synonyms to drop-out.
    - Unbalanced dropout: Characteristics of dropped out patients differed between groups ('informative drop-out'). For example, relatively healthy patients dropped out from the experimental group, whereas patients in relatively poor health dropped out from the control group.
        - *Differences in patient discontinuations over time represent a further limitation.*
- **Underpowered study:** Inability to detect differences between groups due to sample size or insufficient number of outcome events. We are now making this a higher level category since it is a broader concept than sample size and things other than sample size may result in underpowered study e.g. (number of outcome events or measurements with much random error).
    - Sample size: Limitations related to the insufficient number of patients participating in the trial. This may be a result of recruitment difficulties, so "Recruitment less than expected" category is now subsumed by this category.

        - *Among the limitations, our study included only a relatively small sample of the CRASH-2 participants with traumatic brain injury, and a larger sample size could have provided more precise results.*

- **Randomization:** Limitations that have to do with the randomization of patients into different trial arms.
    - Unbalanced groups: After randomization, there were large differences between the groups with respect to (mean values of) prognostically important factors (confounders). This is problematic because the response to interventions may be due to these confounders, rather than the interventions.
        - *In addition the chance imbalance in twin pregnancies between the two arms of the ursodeoxycholic acid comparison made interpretation of endpoints related to prematurity more difficult.*
    - Poor randomization methods: Randomization methods used (e.g., for sequence generation, restriction/stratification, concealment) were not optimal. This also includes a lack of such methods, e.g., that allocation was not concealed (i.e., once a patient is assigned, the next assignment is predictable).
        - *Although open allocation was an unavoidable limitation of the monitoring randomisation.*
- **Blinding:** Limitations related to how the study participants and personnel were blinded to the study groups.
    - *One limitation is that the trial allocation was of necessity open.*
    - Patient: Patients were not blinded with respect to the study groups.
    - Study team: Some people in the study team (investigators, care providers, outcome assessors, statisticians etc.) are not blinded. This replaces the previous more specific categories for Care provider, Data analyst/statistician, and Outcome assessor.

        - *Alternatively, unblinded blood pressures in the clinic reported by general practitioners (albeit using a standardised protocol and automated monitors) may have resulted in an element of bias.*
- **Study Duration:** Limitations that have to do with the length of the study. It could be the experimental phase or the follow-up.
    - Inappropriate duration of experimental phase: The intervention phase is too short. It could be due to early stopping.
        - *Study duration is another potential study limitation given the long half-life of OKZ (31 days).*
    - Inappropriate follow-up duration: Only short term effects were evaluated. Long term (adverse) effects of the interventions were not considered.
        - *Moreover, although 11 years might still not be long enough for deleterious effects on cancer to emerge fully, no adverse trend was noted, even during the later years of post-trial follow-up.*
- **Statistical analysis:** Limitations regarding the methods used for statistical analysis, indicating that the techniques used may not have been appropriate or were suboptimal.
    - Multiple testing: Simultaneous testing of more than one hypothesis. E.g., post-hoc analyses.(https://en.wikipedia.org/wiki/Multiple_comparisons_problem)
        - *We conducted several statistical comparisons and inevitably subgroup analyses involved small numbers; thus power was limited and some significant results could have resulted by chance.*
    - Confounding factors: Findings were not adjusted for covariates.
- **Funding:** The limited or lack of funding affected the study progress or completion.
    - *A randomization ratio with blocks of eight would have been preferable, but it was financially not feasible.*

- **Generalization:** The study results were compromised and may not generalize due to type of setting, specific population, intervention, and measurement instruments.
    - *However, a limitation due to the high-risk nature of our population is that the generalisability of our findings to populations with low-to-moderate risk of vitamin deficiency is unknown.*
- **OTHER**
